# Medical Assistance in Dying: Challenges for Psychiatry

**DOI:** 10.3389/fpsyt.2018.00678

**Published:** 2018-12-10

**Authors:** Roland M. Jones, Alexander I. F. Simpson

**Affiliations:** Complex Care and Recovery Program, Forensic Division, Division of Forensic Psychiatry, Centre for Addiction and Mental Health, University of Toronto, Toronto, ON, Canada

**Keywords:** euthanasia, medically assisted suicide, medical assistance in dying, psychiatry, legislation, Madrid Declaration

A growing number of jurisdictions explicitly exempt doctors from prosecution who assist in or directly cause the death of a person who voluntarily requests it. Medical aid in dying (MAiD) encompasses voluntary euthanasia, where the patient's life is ended by a doctor, and assisted suicide, where the doctor prescribes medication which the person self-administers at a time of their choosing. MAiD in at least one of its forms is permitted in Belgium, Canada, Columbia, Germany, Luxemburg, Netherlands, Switzerland, and seven jurisdictions of the USA (see Table 1), and there is case law, but no federal law that has permitted MAiD in Japan. Switzerland and Germany in some circumstances, allow aid in dying by people other than doctors. Active euthanasia is currently permitted in only five countries, namely Belgium, Netherlands, Luxembourg, Columbia and Canada, and recent legislation enacted permission in Hawaii.

**Table 1:** MAiD provisions in various jurisdictions.

	**Medically Assisted Suicide**	**Active Euthanasia**	**Lower age**	**Permitted for mental conditions**	**Relevant legislation**	**Number per year**	**Comments**
Belgium	Yes	Yes	No lower age limit	Yes	(*Wet Betreffende de Euthanasie, Ministerie Van Justitie, 2002*)	1,807^a^	Intractable and unbearable suffering. Must be terminally ill in the case of children.
Canada	Yes	Yes	18	Unclear	“Bill C-14,” openparliament.ca, June 7, 2016	1,500^e^	For grievous and irremediable medical condition
Columbia	No	Yes	7	No	*Republic of Colombia Constitutional Court, Sentence # c-239/97, Ref. Expedient # D-1490, 1997*.	10–15^e^	IV drugs given by physicians to terminally ill people in hospital to relieve intense pain and suffering. Resolution in 2018 extended to minors.
Germany	Yes	No	Not specified	No	(*German Criminal Code, Section 217*), 2017	Unknown	Assisted Suicide is not illegal if performed on an individual for “altruistic” reasons.
Japan	Not explicit	Not explicit	Not stated	No	“*Tokai- University- Hospital Euthanasia-Case,” Yokohama District Court, 28 March 1995*	Unknown	Unbearable physical pain, death foreseeable, other measures of pain relief exhausted, patient expressed voluntary wish to have life shortened
Luxembourg	Yes	Yes	18	Yes	(*Loi du 16 mars 2009 sur l'euthanasie et l'assistance au suicide, Journal official de Grand-Duche de Luxembourg*)	10^c^	For incurable and terminal diseases that cause physical or psychological constant and unbearable suffering, with no possibility of relief.
Netherlands	Yes	Yes	Newborn; 12 (with parental consent),	Yes	*The Termination of Life on Request and Assisted Suicide (Review Procedures) Act, 2001*.	5,516^b^	Unbearable pain or suffering includes psychological distress, illness must be incurable
Switzerland	Yes	No	18 (25 if not physical suffering)	Yes	(*Swiss Criminal Code, 1937*) (By omission from criminal code, assisted suicide unless for selfish motive is not illegal)	1,004^b^	Another person can assist as long as not for personal gain. Does not have to be a Swiss citizen. Can be assisted by people other than doctors
Victoria (Australia)	Yes	Yes	18	No	Voluntary Assisted Dying Act 2017. Comes into effect on 19 June 2019	n/a	Person must have incurable illness, expected to cause death within 6 months. Person is explicitly not eligible if the only reason is a diagnosis of mental illness.
California	Yes	No	18	No	*ABX2-15 (AB-15), the End of Life Option Act, 2015*	111^c^	Terminal illness that will lead to death in next 6 or 12 months for neurodegenerative condition. Patient must ask 3 times, once in writing, witnessed, and need concurring opinion from another physician that they have < 6 months to live and are of sound mind. ^*^Physician may help administer if the patient is unable to do so
Colorado	Yes	No	18	No	*Proposition 106, the End of Life Options Act, 2016*	50^d^	
District of Columbia	yes	No	18	No	*B21-0038, the Death with Dignity Act of, 2015*	0	
Oregon	Yes	No	18	No	*127.800, et. seq. (The Oregon Death with Dignity Act), 1997*	105^b^	
Hawaii	Yes	Yes^*^	18	No	*Hawaii Revised Statutes 327E-1, et seq.: Uniform Health-Care Decision Act, 2018*	n/a	
Vermont	Yes	No	18	No	*Act 39, Vermont Patient Choice and Control at the End of Life Act, 2013*	13^e^	
Washington	Yes	No	18	No	*Washington Death with Dignity Act, Initiative 1000, RCW 70.245, 2008*	212^c^	
Montana	Yes	No	18	No	*Baxter v. Montana, 2009 MT 449*.	Unknown	Physician's have immunity to prescribe medication that patients self-administer to aid in dying

a During 2014.

b during 2015.

c during 2016.

d during 2017.

e *Approx number per year since introduction*.

The majority of jurisdictions where MAiD is permitted require that the individual is terminally ill, but four jurisdictions have further criteria for MAiD that include a broader range of situations in which a non-terminal disorder causes the person “intractable” or “unbearable pain” or is “a grievous and irredeemable medical condition” (1). What distinguishes the four European countries from the rest of the world (Canada's position is ambiguous in law and is currently subject to debate), is that MAiD can be accessed by people who have non-terminal conditions including mental illness as the primary or only cause of suffering.

MAiD for non-terminal disorders is permitted in Belgium, the Netherlands, Luxembourg, and Switzerland. The literature on its use is small but building. Although psychiatric MAID is relatively uncommon, its use appears to be rising in Belgium and the Netherlands and for a diverse range of disorders and emotional states, including personality disorders and loneliness (2, 3). Concern has been expressed regarding the oversight of the assessment of eligibility, and from the exclusion of family from the process.

Whether MAiD is acceptable and ethically justifiable is controversial, especially so when the person requesting it is not terminally ill (4–6). In addition, there are concerns whether the capacity of a person with mental disorder to request MAiD can be valid for example where depression or psychosis may cause impairment in judgement. Most tests of competence to consent to medical treatment require that the person can “weigh” the information and “appreciate” consequences of their decision (7), yet the ability to do this may be impaired by the effects of mental disorder. Indeed, there are concerns that capacity is not reliably assessed before MAiD (8). Conversely, some view that attitudes are too paternalistic in assuming that people with mental disorder are unable to consent to MAiD (9). The American Psychiatric Association [APA (10)] and the Royal Australian and New Zealand College of Psychiatrists [RANZCP (11)] have considered and rejected the use of MAiD for people who request it for mental disorder. However, the consideration of mental illness as the primary indication for MAiD is likely to be encountered in more jurisdictions.

The WPA has not formally adopted a position on MAiD as applied to psychiatric conditions. The Madrid Declaration (12) however is of relevance, as it addresses euthanasia, stating (emphasis added): *Euthanasia: A physician's duty, first and foremost, is the* ***promotion of health, the reduction of suffering, and the protection of life***. *The psychiatrist, among whose patients are some who are severely incapacitated and incompetent to reach an informed decision, should be particularly careful of actions that could lead to the death of those who cannot protect themselves because of their disability. The psychiatrist should be aware that the views of a patient may be distorted by mental illness such as depression. In such situations, the psychiatrist's role is to treat the illness*.

The Madrid Declaration emphasizes three medical duties: to promote health, relieve suffering and protect life. A number of jurisdictions and medical bodies accept that in terminal illness, when death is imminent (see table), the relief of suffering and respect for a competent wish to die supersedes the ethical duty to protect life. However, in considering MAiD in non-terminal disorders, these duties may be seen to be in conflict with one another. The mental illness that might rarely be seen as meeting end of life care criteria is likely confined to a small group of persons with intractable anorexia nervosa for whom their mental illness can result in serious medical sequelae similar to conditions seen in end of life cases. Outside this, mental illnesses are not terminal (13). Should the suffering caused by symptoms of serious mental illness ever justify MAiD? Should other psychiatric bodies nationally or internationally develop policy positions ahead of such debates? To assist this, we wish to briefly summarize the ethical debates for and against this position.

## Arguments as to Why Mental Illness Might Qualify for MAiD

### Autonomy vs. Paternalism

There is little doubt that serious mental illness can be a painful, irremediable and severe illness that causes great suffering. Treatments may be only partially effective, and may result in side effects intolerable for the person. For some the prognosis for relief of symptoms is poor and, despite symptoms of their illness impairing aspects of their function, the person can still competently evaluate their treatment options and chances of their recovery. Autonomy is the fundamental right for competent adults to self-determination. Under this principle, a competent decision to end one's life in order to relieve one's own suffering, provided it is made rationally and without external influence, is justifiable. Persons with lived experience of serious mental illness may view this as their autonomous wish that physicians should respect, and failure to do so reflects continuing medical paternalism.

### Equivalence With Physical Illness

To differentiate between treatment resistant medical illness and treatment resistant mental illness is a false division, and reflects stigma. Indeed, there is a small but growing literature on “palliative” mental health care that asserts the need to recognize that current treatments, acceptable to the person, will still leave the person suffering and, that as with some physical illnesses, recovery or cure is not obtainable (14).

## Arguments as to Why It Should Not

### Protection of Life Is Paramount

The APA and RANZCP positions on this issue emphasize that there is a profound ethical duty of a doctor that cannot be reconciled with participation in the killing of a patient. That some persons with mental illness will die by suicide is a fact that all practicing clinicians are aware, and there can be respect for the person's wish to suicide, but not for participating in or facilitating it.

Although it is accepted that mental illness may be irremediable in some cases, and doctors have a duty to relieve suffering, they have little ability to predict who will suffer unremittingly and who will recover partially or fully. There are also palliative treatments and other approaches to relieve suffering. For patients, that which seemed to be overwhelming may eventually change and become tolerable. Doctor's opinions as to the patient's prognosis and competence to make this irreversible decision may be prone to error, not based on scientific evidence, and based on the values and moral judgment of the individual the doctor.

### The Slippery Slope

There is a duty for doctors to protect the most vulnerable in society. There is a risk that practices are prone to abuse, and that there is a “slippery slope” of ever more permissive practices that will fail to protect those who are most in need of protection (15). Patients must have confidence in the medical profession, and there is risk of erosion through physician's involvement in roles that may be seen to be at odds with their duty to treat illness and promote health.

## Conclusion

Given the above ethical concerns and rate at which these laws are being considered and reports of some significant problems encountered in countries where it has been adopted, there is a need for psychiatric bodies internationally to consider and provide guidance about how to respond to the question, “Should MAiD extend to serious mental illness?”, and for these questions to debated and carefully considered within the broader psychiatric community.

## Author Contributions

RJ and AS co-authored and edited the manuscript.

### Conflict of Interest Statement

The authors declare that the research was conducted in the absence of any commercial or financial relationships that could be construed as a potential conflict of interest.
